# Supplementary material for: Artificial Intelligence Assisted Computational Tomographic Detection of Lung Nodules for Prognostic Cancer Examination: A Large-Scale Clinical Trial
Source: Biomedicines. 2023 Jan 6;11(1):147. doi: 10.3390/biomedicines11010147 (PMC9856020; doi:10.3390/biomedicines11010147)
Supplement: Supplementary file 1 [file biomedicines-11-00147-s001.zip › biomedicines-2075930-supplementary.pdf]

Supplementary information

Table S1. Summary of Patient Characteristics in Chung Shan Medical University Hospital*.

**Patient characteristics**	Total subjects (N = 100)
**Age (years)**	
Nobs (Missing)[1]	100 (0)
20 ~ 30	2 (2%)
31 ~ 40	2 (2%)
41 ~ 50	11 (11%)
51 ~ 60	39 (39%)
61 ~ 70	43 (43%)
> 70	3 (3%)
**Sex**	
Nobs (Missing)[1]	100 (0)
Female	69 (69%)
Male	31 (31%)
**Ground Truth based on the expert panel**	
Normal subjects	51 (51%)
Subjects with ≥ 1 detected nodule	49 (49%)

[1] Nobs (Missing) = number of observations and missingness.* Patient characteristics are presented as the number of patients and percentage (in parenthesis).

Table S2. Summary of Patient Characteristics in Taipei Veterans General Hospital*.

**Patient characteristics**	Total subjects (N = 100)
**Age (years)**	
Nobs (Missing)[1]	98 (2)
20 ~ 30	1 (1.02%)
31 ~ 40	9 (9.18%)
41 ~ 50	13 (13.27%)
51 ~ 60	34 (34.69%)
61 ~ 70	34 (34.69%)
> 70	7 (7.14%)
**Sex**	
Nobs (Missing)[1]	97 (3)
Female	60 (61.86%)
Male	37 (38.14%)
**Ground Truth based on the expert panel**	
Normal subjects	51 (51%)
Subjects with ≥ 1 detected nodule	49 (49%)

[1] Nobs (Missing) = number of observations and missingness. * Patient characteristics are presented as the number of patients and percentage (in parenthesis).

Table S3. Nodule counts and size distribution in Chung Shan Medical University Hospital*.

**Nodule size**	Total nodules (n = 107)
**Nodule diameter (mm)**	
4 ~ < 5	14 (13.08%)
5 ~ < 6	30 (28.04%)
6 ~ < 7	18 (16.82%)
7 ~ < 8	13 (12.15%)
8 ~ < 9	6 (5.61%)
9 ~ < 10	4 (3.74%)
10 ~ < 11	5 (4.67%)
11 ~ < 12	3 (2.8%)
12 ~ < 13	2 (1.87%)
13 ~ < 14	2 (1.87%)
14 ~ < 15	5 (4.67%)
15 ~ < 16	2 (1.87%)
16 ~ < 17	3 (2.8%)

* Distribution of categorical nodule size is presented as the number of nodules and percentage (in parenthesis).

Table S4. Nodule counts and size distribution in Taipei Veterans General Hospital*.

**Nodule size**	Total nodules (n = 98)
**Nodule diameter (mm)**	
4 ~ < 5	11 (11.22%)
5 ~ < 6	14 (14.29%)
6 ~ < 7	10 (10.2%)
7 ~ < 8	9 (9.18%)
8 ~ < 9	12 (12.24%)
9 ~ < 10	15 (15.31%)
10 ~ < 11	7 (7.14%)
11 ~ < 12	1 (1.02%)
12 ~ < 13	1 (1.02%)
13 ~ < 14	2 (2.04%)
14 ~ < 15	3 (3.06%)
16 ~ < 17	1 (1.02%)
17 ~ < 18	1 (1.02%)
18 ~ < 19	2 (2.04%)
19 ~ < 20	1 (1.02%)
21 ~ < 22	2 (2.04%)
23 ~ < 24	2 (2.04%)
24 ~ < 25	2 (2.04%)
28 ~ < 29	1 (1.02%)
29 ~ < 30	1 (1.02%)

* Distribution of categorical nodule size is presented as the number of nodules and percentage (in parenthesis).

Table S5. Slice thickness and CT brand of the current study in Chung Shan Medical University Hospital*.

**Imaging characteristics**	Total subjects (N = 100)
**Slice thickness (mm)**	
0.5 < ~ < 1	21 (21%)
1	26 (26%)
2 ~ 3	53 (53%)
**Manufacturer**	
GE Medical Systems	12 (12%)
Siemens	78 (78%)
Toshiba	10 (10%)

* Imaging characteristics are presented as the number of subjects and percentage (in parenthesis).

Table S6. Slice thickness and CT brand of the current study in Taipei Veterans General Hospital.

**Imaging characteristics**	Total subjects (N = 100)
**Slice thickness (mm)**	
0.5 < ~ < 1	4 (4%)
1	71 (71%)
1 < ~ < 2	9 (9%)
2 ~ 3	16 (16%)
**Manufacturer**	
GE Medical Systems	13 (12%)
Siemens	55 (55%)
Toshiba	3 (3%)
Philips	29 (29%)

Table S7. CT machine model type used in the current study.

**Manufacturer brand**	**Model type**
GE MEDICAL SYSTEMS	LightSpeed VCT
GE MEDICAL SYSTEMS	Optima CT660
GE MEDICAL SYSTEMS	Revolution Apex
GE MEDICAL SYSTEMS	Revolution CT
GE MEDICAL SYSTEMS	Revolution EVO
GE MEDICAL SYSTEMS	Revolution HD
SIEMENS	Emotion 16 (2010)
SIEMENS	Sensation Cardiac 64
SIEMENS	SOMATOM Definition AS
SIEMENS	SOMATOM Definition AS+
SIEMENS	SOMATOM Definition Flash
SIEMENS	SOMATOM Force
SIEMENS	syngo.via.VB20A
Philips	iCT 256
TOSHIBA	Alexion
TOSHIBA	Aquilion
TOSHIBA	Aquilion ONE

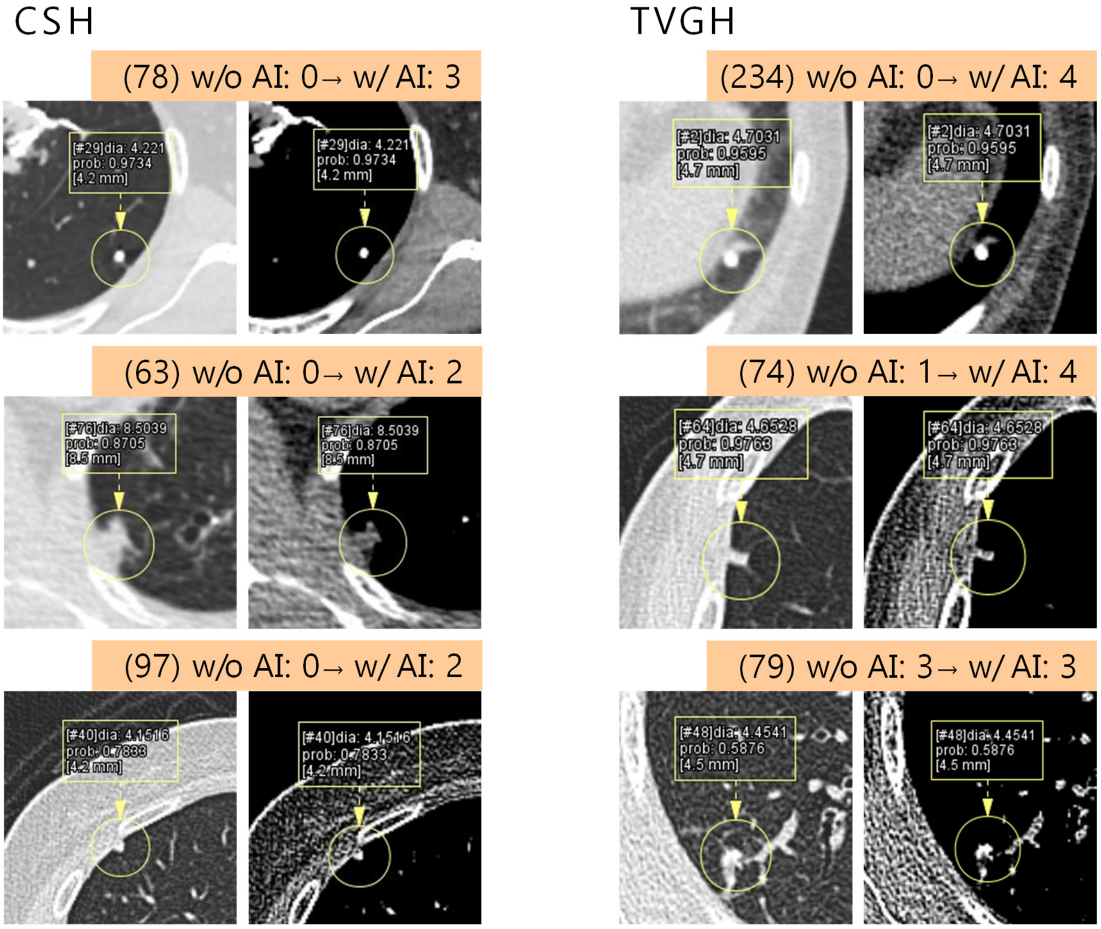

Figure S1. Solid nodule detection ameliorated by AI assistance from 4 doctors in CSH TVGH, respectively. The number in the () represented the CT slice in the clinical trials. Data representation such as "w/o AI: 0→ w/ AI: 3" means the nodule detected by 0/4 doctors in the scenario without AI assistance increases to 3/4 doctors detected the identical nodule in the scenario with AI assistance. Two columns show identical CT images with distinct grey-leveled contrast. CSH: Chung Shan Medical University Hospital; TVGH: Taipei Veterans General Hospital

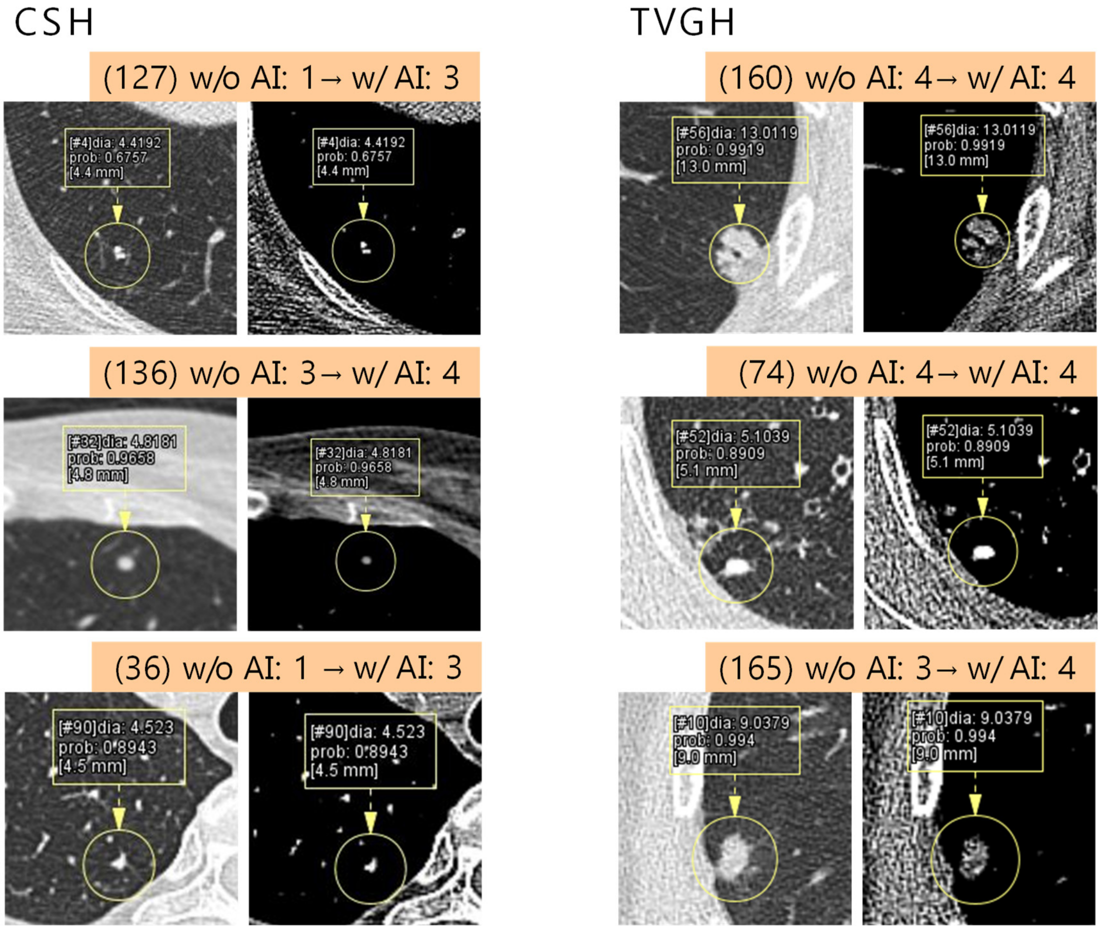

Figure S2. Solid nodule detection ameliorated by AI assistance from 4 doctors in CSH and TVGH, respectively. The number in the () represented the CT slice in the clinical trials. Data representation such as "w/o AI: 1→ w/ AI: 3" means the nodule detected by 1/4 of the doctors in the scenario without AI assistance increases to 3/4 doctors detected the identical nodule in the scenario with AI assistance. Two columns show identical CT images with distinct grey-leveled contrast. CSH: Chung Shan Medical University Hospital; TVGH: Taipei Veterans General Hospital
